# Correction: Impact of Pregnancy on Intra-Host Genetic Diversity of Influenza A Viruses in Hospitalised Women: A Retrospective Cohort Study. *J. Clin. Med.* 2020, *9*, 1974

**DOI:** 10.3390/jcm9010058

**Published:** 2019-12-25

**Authors:** Gregory Destras, Maxime Pichon, Bruno Simon, Martine Valette, Vanessa Escuret, Pierre-Adrien Bolze, Gil Dubernard, Pascal Gaucherand, Bruno Lina, Laurence Josset

**Affiliations:** 1 Virpath, INSERM U1111, CNRS UMR5308, International Center for Infectiology Research, ENS Lyon, Claude Bernard Lyon 1 University, 69008 Lyon, France; gregory.destras@chu-lyon.fr (G.D.); maxime.pichon@chu-poitiers.fr (M.P.); vanessa.escuret@chu-lyon.fr (V.E.); bruno.lina@chu-lyon.fr (B.L.); 2 Virology Laboratory, Infectious Agents Institute, CBN, Groupement Hospitalier Nord, Hospices Civils de Lyon, 69004 Lyon, France; sib0.smb@gmail.com (B.S.); martine.valette@chu-lyon.fr (M.V.); 3 Centre National des Virus des infections Respiratoires, Infectious Agents Institute, CBN, Groupement Hospitalier Nord, 69004 Lyon, France; 4 Service de Chirurgie Gynécologique et Oncologique—Obstétrique, Centre Hospitalier Lyon Sud, Hospices Civils de Lyon, 69310 Pierre-Bénite, France; pierre-adrien.bolze@chu-lyon.fr; 5 Hospices Civils de Lyon, Service de Gynécologie et d’Obstétrique, Hôpital de la Croix Rousse, 69004 Lyon, France; gil.dubernard@chu-lyon.fr; 6 Consultation Obstétrique, Groupement Hospitalier Est, Hospices Civils de Lyon, 69500 Bron, France; pascal.gaucherand@chu-lyon.fr

The authors wish to make the following corrections to this paper [1].

There was an error in Figure 1: dots describing the ihSNVs count for each patient in panels A and B were omitted during figure formatting, thus Figure 1 needs to be corrected: 
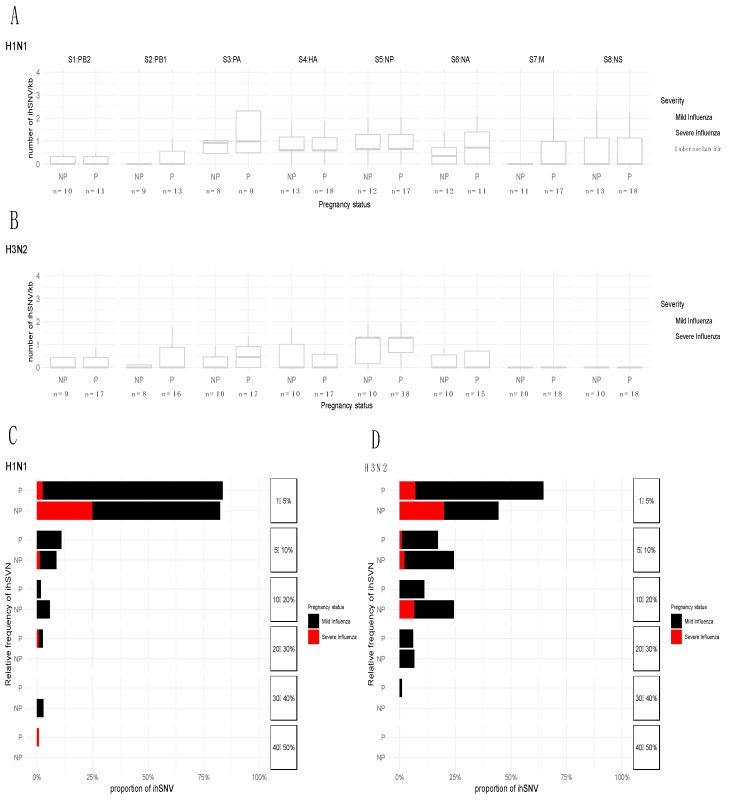
 and should be replaced with 
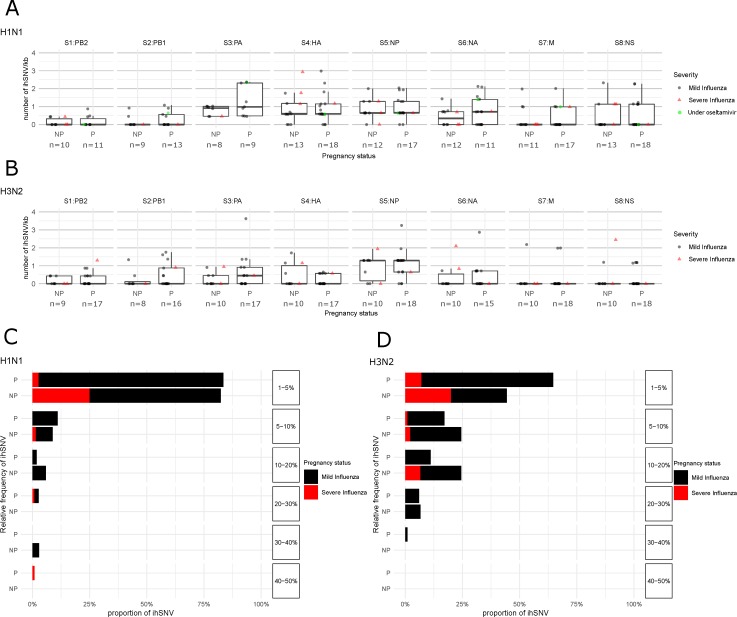


The authors apologize to the readers for any inconvenience caused by these changes. It is important to state that this correction does not affect our study’s results and involve no changes or modifications in the original data supporting our results. The original manuscript will remain online on the article webpage, with reference to this Correction.
