# Supplementary material for: Impact of Pregnancy on Intra-Host Genetic Diversity of Influenza A Viruses in Hospitalised Women: A Retrospective Cohort Study
Source: J Clin Med. 2019 Nov 14;8(11):1974. doi: 10.3390/jcm8111974 (PMC6912736; doi:10.3390/jcm8111974)
Supplement: Supplementary file 1 [file jcm-08-01974-s001.zip › supplementary/SupplementaryFiles.docx]

**Supplementary Materials**

**Tables**

**Table S1.**  Sequencing performance.

|  | Pregnant women  n=36 | Non  -pregnant women  n=23 | p |
| --- | --- | --- | --- |
| H3N2, median of mean depth of coverage per sample |  |  |  |
| S1 | 22365 | 30233 | 0.10 |
| S2 | 12458 | 12743 | 1.00 |
| S3 | 15481 | 19665 | 0.30 |
| S4 | 20668 | 19725 | 0.60 |
| S5 | 40785 | 38532 | 1.00 |
| S6 | 15588 | 15047 | 1.00 |
| S7 | 57320 | 46165 | 0.30 |
| S8 | 24700 | 22844 | 0.60 |
| H1N1, median of mean depth of coverage per sample |  |  |  |
| S1 | 3741 | 5164 | 0.20 |
| S2 | 3905 | 5625 | 0.20 |
| S3 | 2709 | 2333 | 0.90 |
| S4 | 14362 | 26140 | 0.06 |
| S5 | 35182 | 41263 | 0.40 |
| S6 | 17715 | 22427 | 0.20 |
| S7 | 45576 | 66754 | 0.30 |
| S8 | 41650 | 50939 | 0.10 |

p: p-value.

**Table S2.** Prevalence and putative functions of non-synonymous ihSNV.

| **H3N2** | **Non-synonymous variants** | **Pregnancy status (n)** | **Severity associated (n)** | **protein domains** |
| --- | --- | --- | --- | --- |
| PB2 | H110L | P (1) | NS | N1 domain |
|  | A274T | P (1) | NS | Mid domain |
|  | R389K | P (1) | S (1) | Cap-binding |
|  | A395S | P (1) | NS | Cap-binding |
|  | Y658H | P (1) | NS | 627 domain |
|  | T711S | P (1) | NS | NLS |
|  | I754F | P (1) | S (1) | NLS |
| PB1 | V114I | NP (1) | NS | Fingers |
|  | **K279Q** | P (4) | S (1) | Palm |
|  | **Q460K** | P (3) | NS | Palm |
|  | F574L | P (1) | NS | Thumb |
|  | T570A | P (1) | NS | Thumb |
|  | K577R | P (1) | NS | Thumb |
|  | A652T | P (1) | S (1) | Priming-loop |
|  | S665P | P (1) | NS | Thumb |
| PA | V62M | P (1) | NS | Endonuclease |
|  | L65F | P (1) | NS | Endonuclease |
|  | V127F | P (1) | NS | Endonuclease |
|  | E416G | P (1) | NS | C-terminal domain |
|  | I423M | P (1) | NS | C-terminal domain |
|  | V450A | P (1) | NS | C-terminal domain |
|  | R551K | NP (1) | NS | C-terminal domain |
|  | M595L | NP (1) | S (1) | C-terminal domain |
|  | R638M | P (1) | NS | C-terminal domain |
|  | L640V | P (1) | NS | C-terminal domain |
| HA | T25A | P (1) | NS | HA1 |
|  | N122K | NP (1) | S (1) | HA1 |
|  | S150N | NP (1) | S (1) | HA1 |
|  | Q364K | P (1) | NS | HA2 |
|  | D514N | NP (1) | NS | HA2 |
| NP | G5S | NP (1) | S (1) | NLS |
|  | A85V | P (1) | NS | PB2 binding domain |
|  | G169C | P (1) | NS | - |
|  | L256P | P (1) | NS | NP binding domain |
|  | A451T | NP (1) | S (1) | NP and PB2 binding domain |
| NA | I26T | P (1) | NS | transmembrane domain |
|  | E59K | NP (1) | NS | stalk domain |
|  | Y84H | P (1) | NS | stalk domain |
|  | V445M | P (1) | NS | head domain |
| M | -- |  | NS |  |
| NS | D34G | NP (1) | NS | RNA-binding domain |
|  | S42P | P (1) | NS | RNA-binding domain |
|  | V60I | P (1) | NS | RNA-binding domain |
|  | E66K | NP (1) | S (1) | RNA-binding domain |
|  |  |  |  |  |
|  |  |  |  |  |
| **H1N1** |  |  |  |  |
| PB2 | G388R | P (1) | NS | Cap-binding |
|  | D390E | P (1) | NS | Cap-binding |
|  | V547I | NP (1) | NS | 627 domain |
| PB1 | **K279Q** | P (4) + NP (1) | NS | Palm |
|  | **Q460K** | P (1) | NS | Palm |
| PA | E493A | P (9) + NP (8) | NS | C-terminal domain |
|  | G555S | P (1) | NS | C-terminal domain |
|  | M561I | P (1) | NS | C-terminal domain |
| HA | I116M | P (1) | NS | HA1 |
|  | S122P | P (1) | NS | HA1 |
|  | **P182Q** | P (1) + NP (4) | NS | HA1 |
|  | A186T | P (1) | NS | HA1 |
|  | T241A | NP (1) | NS | HA1 |
|  | D269N | NP (1) | NS | HA1 |
|  | A305V | P (1) | NS | HA1 |
|  | W341R | P (1) | NS | HA2 |
|  | W341C | P (1) | NS | HA2 |
|  | **T342A** | P (2) + NP (2) | S (1) | HA2 |
|  | **E356K** | P (9) + NP (3) | NS | HA2 |
|  | N450S | P (1) | NS | HA2 |
|  | D500S | NP (1) | S (1) | HA2 |
| NP | **T130K** | P (13) + NP (9) | NS | PB2 binding domain |
|  | R317G | NP (1) | NS | NP binding domain |
|  | S326G | P (1) | NS | NP binding domain |
|  | V414M | P (1) | NS | NP binding domain |
|  | F458L | P (1) | NS | NP binding domain |
|  | N483T | NP (1) | NS | NP binding domain |
| NA | G11S | P (1) | S (1) | transmembrane domain |
|  | I29T | P (1) | NS | transmembrane domain |
|  | Q51R | P (1) | NS | stalk domain |
|  | A86T | P (1) | NS | stalk domain |
|  | V94I | NP (1) | NS | head domain |
|  | R156L | P (1) | NS | head domain |
|  | S200G | P (1) | NS | head domain |
|  | P272H | P (1) | NS | head domain |
|  | N325S | NP (1) | NS | head domain |
|  | N341D | NP (1) | NS | head domain |
|  | I393T | P (1) | NS | head domain |
|  | R430L | P (1) | NS | head domain |
|  | T466I | P (1) | NS | head domain |
| M | -- |  | NS |  |
| NS | N25S | NP (1) | S (1) | RNA-binding domain |
|  | E66K | P (1) | NS | RNA-binding domain |
|  | E72K | P (1) | NS | RNA-binding domain |
|  | M93I | NP (1) | NS | Effector domain |
|  | N139S | P (1) | NS | Effector domain |
|  | F150L | NP (1) | S (1) | Effector domain |
|  | E153G | P (1) | NS | Effector domain |

**Figures**

**Figure S1.** Segment coverage of H1N1 and H3N2 influenza virus genomes. Mean depth line for each segment is framed by grey lines corresponding to the first and third quartiles of depth of coverage.

**PB2**

**PB1**

**PA**

**HA**

**NP**

**NA**

**M1**

**NS1**

**Figure S2–S9.** Phylogenetic tree of PB2, PB1, PA, HA, NP, NA, M1, NS1 for H1N1 (A) and H3N2 viruses (B); Phylogenetic trees were generated using Seaview software, according to BioNeighbour-Joining distance Method and Poisson correction.

**Figure S10.** Comparison of IAV non-synonymous (NS) ihSNV between pregnant and non-pregnant women. Number of non-synonymous ihSNV/kb per segment (S1 to S8) according to pregnancy status (P: pregnant; NP: non-pregnant) for H1N1 (A) and H3N2 (B) viruses. Each point represents a patient, with black dot for mild influenza and red triangles for severe influenza. Distribution of non-synonymous ihSNV relative frequencies according to pregnancy status (P: pregnant; NP: non-pregnant) for H1N1 (C) and H3N2 (D) viruses. Severe influenza is in red.
